# Internet Access as an Essential Social Good

**DOI:** 10.1007/978-3-030-65355-2_4

**Published:** 2021-03-20

**Authors:** Alfred Archer, Nathan Wildman

**Affiliations:** 1 Tilburg University, Tilburg, The Netherlands; 2 Tilburg University, Tilburg, The Netherlands; 3 Tilburg University, Tilburg, The Netherlands; 4 Tilburg University, Tilburg, The Netherlands; Department of Philosophy, Tilburg School of Humanities and Digital Sciences, Tilburg, The Netherlands

## Abstract

During the coronavirus crisis, educational activities and nearly all social contact with friends and family were conducted via online communication tools. Such tools can only be used effectively if an individual has suitable internet access. Thankfully, the Netherlands is one of the EU leaders when it comes to Next Generation Access (NGA) coverage, with 98% of Dutch households having access to these high-speed connections; this is well above the USA (94%) and EU (87%) averages. However, this still means that nearly 344,000 individuals living in the Netherlands lack a strong internet connection. Here, we contend that the coronavirus crisis, and especially the associated lockdown wherein individuals were strongly encouraged to not leave their homes, has made it clear that high-speed internet access is a necessary good for modern social living.

During the coronavirus crisis, educational activities and nearly all social contact with friends and family were conducted via online communication tools. Such tools can only be used effectively if an individual has suitable internet access.

Thankfully, the Netherlands is one of the EU leaders when it comes to Next Generation Access (NGA) coverage,^1^ with 98% of Dutch households having access to these high-speed connections; this is well above the USA (94%) and EU (87%) averages.^2^ However, this still means that nearly 344,000 individuals living in the Netherlands lack a strong internet connection.

Here, we contend that the coronavirus crisis, and especially the associated lockdown wherein individuals were strongly encouraged to not leave their homes, has made it clear that high-speed internet access is a necessary good for modern social living.

## Internet Access as a Pragmatic Necessity for Other Rights

One key reason for thinking that internet access is an essential social good is because it is “pragmatically essential” for protecting, promoting, and in many cases, exercising certain human rights (Reglitz 2020: 316).

Consider the right to freedom of expression—that is, the right to “seek, receive, and impart information and ideas of all kinds, regardless of frontiers” (International Covenant on Civil and Political Rights, Article 19, §2). Exercising this right essentially involves having a platform for putting forward one’s ideas in a public sphere. Pre-crisis, this could be done via some broadly physical means, or online. The crisis, and in particular the subsequent lockdown, effectively eliminated the option of doing so physically—for public health reasons, all public discourse shifted onto the internet. In this way, internet access became necessary to effectively exercise one’s right to free expression.

Similarly for the right to assembly: once we were unable to physically congregate, the only means of properly exercising this right was via online association.

More generally, suitable internet access looks like a prerequisite for engaging in political life during the coronavirus crisis. Consequently, not having suitable internet access “excludes people from the forums and platforms in which much of today’s political debate takes place, and in which most of the politically relevant information is shared” (Reglitz 2020: 320). The upshot is that a properly functioning post-crisis democracy requires citizens to have viable access to the internet.

The status of internet access as an essential social good is illustrated by reports from organizations that aid refugees. The UNHCR, for instance, reports that refugees, some of the world’s most vulnerable people in desperate need of life’s basic needs, find internet access absolutely essential (UNHCR 2016). This is because the internet allowed them to communicate with loved ones from whom they were separated and let each other know if they were safe. As one aid worker noted, “What we are hearing is that technology [internet] is regarded by the people we are here to serve as a need as important as food or clothes” (UNHCR 2016). The UNHCR goes on to note the critical role of internet access in enabling refugees to exercise a right to education, health care, and to work via online entrepreneurship opportunities.

Recognizing the connection between internet access and the exercise of certain rights may have implications for thinking about how access is managed for certain groups. For example, prisoners in the Netherlands have limited access to the internet. This access is driven by an interest in providing entertainment and educational opportunities (Tighe 2016). If internet access is thought of as an essential social good, however, this would give reason to revise this attitude: internet access would not be a luxury or a perk but a necessity for exercising certain rights, particularly given the physical restrictions prisoners are subject to.

## Poverty

Thinking about the nature of poverty gives us another important reason to think that internet access is an essential social good. In his discussion about taxation in The Wealth of Nations (1776), Adam Smith distinguishes between two kinds of goods: necessaries and luxuries. Notably, he claimed that necessities include not only those goods that are needed for basic survival, such as food, water, and shelter but also “whatever the custom of the country renders it indecent for creditable people [...] to be without” (Smith 1776, Book V, Chap. 2, part II, article 4). That is, necessaries as those goods that people would be ashamed to be seen in public without.

Building on this, sociologists like Peter Townsend (1962) have claimed that poverty should be understood in relation to a particular society at a particular time; i.e., poverty involves lacking the resources needed to have a social life in your contemporary society.

While in Smith’s society (eighteenth Century Scotland) these goods included leather shoes and linen shirts, in contemporary western societies, internet access is plausibly one such necessary: many of those without internet access are likely to be ashamed or embarrassed to admit their deprivation. Further, as noted above, during the lockdown, internet access is not only needed to appear in public without shame but is necessary to appear in public at all. More generally, if internet access is needed to have a social life in contemporary western societies, then those who cannot afford internet access should be counted as living in poverty.

## Social Deprivation

Additionally, the need for internet access to participate in social life during lockdown is itself a reason to think that internet access is an essential social good. According to philosopher Kimberley Brownlee (2013), the right not to experience social deprivation should be accepted as a human right. As social creatures, human beings need social interaction in order to have a minimally decent life. Being deprived of decent social interaction leads to lower well-being, poorer mental, and physical health, and in extreme cases, amounts to a form of torture. For people living alone during lockdown, internet access was the only option available for social interaction and so was essential for protecting human right against social deprivation. Of course, this was (hopefully) a temporary situation. However, as more and more social interaction takes place online, those without internet access will find themselves locked out from an increasing number of social spaces. Providing internet access is needed, then, to protect people’s rights against social deprivation.

## Education

Finally, unequal access to the internet also runs the risk of making existing educational inequalities worse. During periods of lockdown, the vast majority of educational activities moved online. A significant proportion of these will continue to be online for the foreseeable future. Those who lack good quality internet access will be disadvantaged by this and, unfortunately, these are often students who already face other educational disadvantages. All students must be given good quality internet access, then, to help prevent the widening of these educational inequalities. This will be especially important for as long as schools and universities continue to deliver much of their education online but will continue to be an important issue beyond this point as well.

## Conclusion

In the above, we have argued that, without suitable internet access, individuals are unable to properly exercise a number of their fundamental human rights, cannot fully participate in democratic political institutions, can (arguably) be said to be living in poverty, and are being made to suffer from social deprivation. Further, unequal access exacerbates educational inequalities. Taken together, these reasons make a strong case for thinking that internet access is an essential social good. And, as a knock-on consequence, governments have a clear responsibility to ensure that citizens have suitable access, e.g., via nationalization^3^ or sufficient regulation.

## Footnotes

1 Defined to include technologies like FTTH, FTTB, Cable Docsis 3.0, VDSL, or other broadband connections of at least 30 Mbps downloads.

2 European Commission Report on Digital Economy and Social Index 2020, https://ec.europa.eu/newsroom/dae/document.cfm?doc_id=67079 (accessed July, 01, 2020); FCC eighth Broadband Progress Report, https://www.fcc.gov/reports-research/reports/broadband-progress-reports/eighth-broadband-progress-report (accessed July, 01, 2020).

3 For example, in the UK, the Jeremy Corbyn-led Labour Party set out plans to ensure UK-wide high-speed internet access by nationalizing BT and creating a new British Broadband public service. Labour claimed that this could ‘boost productivity by £59 billion by 2025; bring half a million people back into the workforce; and boost rural economies, with an estimated 270,000 people more able to move to rural areas’ (Labor Party press release November 14, 2019, https://labour.org.uk/press/british-broadband-labour-sets-out-mission-to-connect-communities-across-britain-by-delivering-free-full-fibre-broadband-for-all/, accessed on July, 27, 2020).
